# Application of Cryopreserved Fibroblast Culture with Au Nanoparticles to Treat Burns

**DOI:** 10.1186/s11671-016-1242-y

**Published:** 2016-01-14

**Authors:** Nataliia Volkova, Mariia Yukhta, Olena Pavlovich, Anatoliy Goltsev

**Affiliations:** Laboratory of Biotechnology and Applied Nanotechnology, Department of Cryobiology of Reproductive System, Institute for Problems of Cryobiology and Сryomedicine of the National Academy of Sciences of Ukraine, Pereyaslavskaya Str., 23, Kharkov, 61015 Ukraine

**Keywords:** Cells, Culture, Fibroblasts, Gold nanoparticles, Experimental burns, Regeneration

## Abstract

The aim was to investigate a possibility of using the cryopreserved human culture of fibroblasts (CrHFC) with gold nanoparticles (AuNPs) to treat experimental burns in rats.

The third-degree burns were modeled in white male rats. All the animals with burns were divided into three experimental groups: control group with no wound treatment; group 1 was composed of animals with CrHFC application; and group 2 consisted of those with CrHFC and AuNPs (6 μg/ml) application to a burn surface the next day after the injury. The CrHFC was applied to the methylcellulose gel in a dose of 5 × 10^4^ of viable cells per 1 cm^2^ of the burn. The animals were removed from the experiment on day 21 after the treatment.

The CrHFC use alone and with AuNPs to the surface of burns stimulated the wound healing compared with the control. The effect of using CrHFC was less pronounced compared to the CrHFC application with AuNPs. It was reflected in a slower recovery of burns and moderate lymphocytic infiltration of granulation tissue. Immunofluorescent analysis emphasized that the use of CrHFC with AuNPs accelerated the skin synthetic processes and was helpful in recovering type I and III collagen content on day 21 after therapy.

The results were likely related primarily to the unique structure and antimicrobial properties of AuNPs. Our experimental study of the effect of CrHFC with AuNPs application on regenerative processes in burns gives some pre-conditions to the following advanced bio- and nanotechnology developments.

## Background

One of the urgent cell biology tasks is to study the effect of stem cells on regeneration and reparation of damaged tissues. Worldwide, in the practice of many burn centers along with traditional methods of the treatment of burn wounds, the modern biotechnological approaches are used [1]. A wound repair is a complicated and dynamic process which comprises of inflammation, angiogenesis, and tissue formation/remodeling [2]. The use of cultured cells derived from the patient’s own tissue (skin, bone marrow, fat, and other sources) is the most promising direction in burn treatment because they do not cause an immune rejection [3, 4]. The fibroblasts cultured beyond the body for therapeutic purposes were attempted to be used just after establishing the fact that the normal fibroblasts retained a diploid karyotype in the culture without an oncogenic potential and had a limited survival time and low expression of histocompatibility antigens [5]. The low temperature storage provides an essential number of available cells at any time as well as it does not affect the morphological and functional states of human fibroblasts due to the development of effective cryopreservation programs [6]. Usually, fibroblasts activate reparative processes in a paracrine manner by secreting into the environment a large number of biologically active substances, among which are various growth factors, cytokines, extracellular matrix components, and enzymes [7].

The gold nanoparticles (AuNPs) as a structural basis of nanocomposites have been previously applied to deliver molecules into cells with a therapeutic effect. In all these cases, AuNPs enter inside a human body and contact the cells. Nanocompounds in high concentrations can have a toxic effect on the cells in vitro and in vivo. Unfortunately, there are no clear conclusions about the variability of nanoparticle parameters such as physical and chemical properties as well as about cell study conditions [8]. In some reports, there are contradictory data about AuNP application during cell culturing both in monolayer and suspension. Early, we studied the viability, proliferative capacity, and apoptosis/necrosis in human fibroblast culture (HFC) prior to and after cryopreservation in the presence of gold nanoparticles [9]. The use of AuNPs under low concentrations resulted in an increased proliferative activity of HFC with no activation of apoptosis and necrosis. Afterwards, it was an actual task to study the possibility of cryopreserved human fibroblast application with AuNPs to stimulate the regeneration in vivo. For this purpose, a model of thermal burns in rats was chosen as an experimental pathology. The research hypothesis was based on the regenerative effect of HFC and antioxidant properties of AuNPs.

## Methods

### Animals and Ethics Statement

The experiments were performed in 15 outbreed male white rats weighing 210 ± 20 g (mean ± standard deviation). All the rats were housed in plastic cages (one animal per cage) and kept at a controlled temperature (18–22 °C), humidity (30–70 %), and lighting (lights on from 8 a.m. to 8 p.m.) on a standard diet with free access to food and water. The rats were acclimated for at least 7 days prior to the experiments. During housing, the animals were daily monitored for a health status. No adverse events were observed. All the manipulations were carried out in a strict accordance with the requirements of the “European Convention for the Protection of Vertebrate Animals used for Experimental and other Scientific Purposes”. The protocol was approved by the Committee on the Ethics of Animal Experiments of the Institute for Problems of Cryobiology and Сryomedicine of the National Academy of Sciences of Ukraine (Permit No. 2014-02).

### Culture and Cryopreservation of Human Fibroblasts

Human fibroblasts were cultured in plastic flasks in DMEM (Sigma, USA) supplemented with 5 % fetal bovine serum (FBS) (*v*/*v*) (HyClone, USA), gentamicin (150 mg/ml) (Farmak, Kiev, Ukraine), and amphotericin B (5 μg/ml, PAA). The seeding density was 1.2 × 10^3^ cells/cm^2^. The cells were cultured in the incubator (Sanyo, Japan) under 37 °С with 5 % СО_2_ in humid atmosphere. The culture medium was replaced every 3 days. The cells were passaged at 80–90 % confluence. Human fibroblasts expressed growth properties and during serial passages preserved the initial morphological structure of monolayer without evidences of cell degeneration.

The HFC was cryopreserved under protection of 10 % DMSO (PanEko, Russia) and 20 % FBS on the nutritive medium base. Freezing was performed at 1 °C/min to −80 °C, followed by plunging into liquid nitrogen. The samples were thawed on water bath at 40 °C up to the liquid phase appearance [10]. Cryoprotectant was removed by slowly adding a tenfold volume of Hanks’ solution (PAA) followed by centrifugation at 834 × *g* for 5 min.

### Manipulations with AuNPs

AuNPs were obtained by citrate synthesis [11] with an initial metal concentration of 45 μg/ml. The average size of AuNPs was 15 nm. They were entered into the human fibroblasts after thawing by a passive diffusion during 1-h incubation at 37 °С in the nutritive medium supplemented with AuNPs (6 μg/ml).

After incubation, apoptotic and necrotic processes in cells were investigated with FACSCalibur using annexin V (BD, USA) and 7-amino-actinomycin D (7AAD) (BD, USA) dyes. The results were analyzed with WinMDI v.2.8 software. The effect of AuNPs in 6 μg/ml concentration on a proliferative ability of cryopreserved human fibroblasts was evaluated during 7 days of culturing. Cryopreserved human fibroblasts cultured in nutritive medium with no addition of AuNPs served as the control. The proliferative dynamics was investigated on days 3, 5, and 7 of cultivation by enzymatic removal out of a plastic and counting the cell number by a traditional method in Goryaev’s chamber.

### Study Design

Thermal burns grade 3 were modeled with a special stainless steel device with a 5.4-cm^2^ operational area and equipped with a control thermometer [12]. Before injuring, the hair was removed from the area of around 8 cm^2^ on the left dorsal part of the back. An applicator heated up to 100 °C was applied to this area for 5 s. For all the manipulations, the animals were anesthetized by an intraperitoneal injection of ketamine (10 mg/kg, Biolik, Kharkov, Ukraine) combined with xylazine (1 mg/kg, Bioveta, Prague, Czech Republic). During the 5-day postoperative period, the animals received ketofen (2 mg/kg, Merial SAS, Lyon, France) for anesthesia.

On the following day after burn injuring, the animals were randomly divided into three groups (*n* = 5): control group with naturally occurred healing (without treatment); research group 1 with cryopreserved HFC (CrHFC) application to the burn surface; and research group 2 with CrHFC with AuNP (6 μg/ml) application to the burn surface. The cell application was carried out in the methylcellulose gel in a dose of 5 × 10^4^ per 1 cm^2^ of burn surface.

### Evaluation Methods

For all animals, a clinical observation was daily performed; the body weight and area of the burn were studied on days 4, 7, 14, and 21. The animals were removed out of the experiment on day 21 after treatment.

For histological studies, the skin samples were fixed in 10 % neutral formalin aqueous solution and coded by a third person who was not involved in the experiment to maintain the blinding. Serial paraffin sections of the skin were done with a 4–5-μm thickness and stained with hematoxylin and eosin.

An assessment of type I and III collagen content was performed with cryostat sections of the skin (7 μm) using monoclonal antibodies to type I collagen (1:2000, COL-1, Sigma-Aldrich, USA) with CFTM488A (Sigma-Aldrich, USA) and monoclonal antibodies to type III collagen (1:80, Millipore, USA) with goat anti-rat IgG Alexa fluore 647 conjugate (Millipore, USA) according to the manufacturer’s instructions. The fluorescent microscopy of the skin sections was performed by a laser scanning microscope LSM 510 META (Carl Zeiss, Germany). Autofluorescence was quenched by 0.3 M glycine solution (PAA, Austria) for 20 min [13].

### Statistical Analysis

The results were processed with a non-parametric Mann-Whitney *U* test using “Statistica 8” program. The results were presented as mean ± standard deviation. *p* < 0.05 was considered statistically significant.

## Results

### Characteristics of CrHFC

The cytofluorimetric results showed that AuNPs in the 6 μg/ml concentration did not cause the development of apoptosis/necrosis in CrHFC after 1-h incubation (Table 1).

**Table 1:** Cytofluorimetric analysis of CrHFC after 1 h incubation with AuNPs, staining with annexin V and 7AAD

Group	Аnnexin V^+^/7AAD^−^	Аnnexin V^−^/7AAD^−^	Аnnexin V^+^/7AAD^+^ + annexin V^−^/7AAD^+^
CrHFC	1.4 ± 0.7	83.8 ± 1.0	16.8 ± 1.2
CrHFC + AuNPs (6 μg/ml)	1.6 ± 0.6	80.1 ± 1.2	18.3 ± 1.3

In all the groups, the fibroblasts adhered after 2 h of culturing, and 1 day later, most of the cells were spindle-shaped with well-defined borders. The morphology of cells cultivated with AuNPs did not differ from that of the control samples during all the observation periods.

**Fig. 1:**
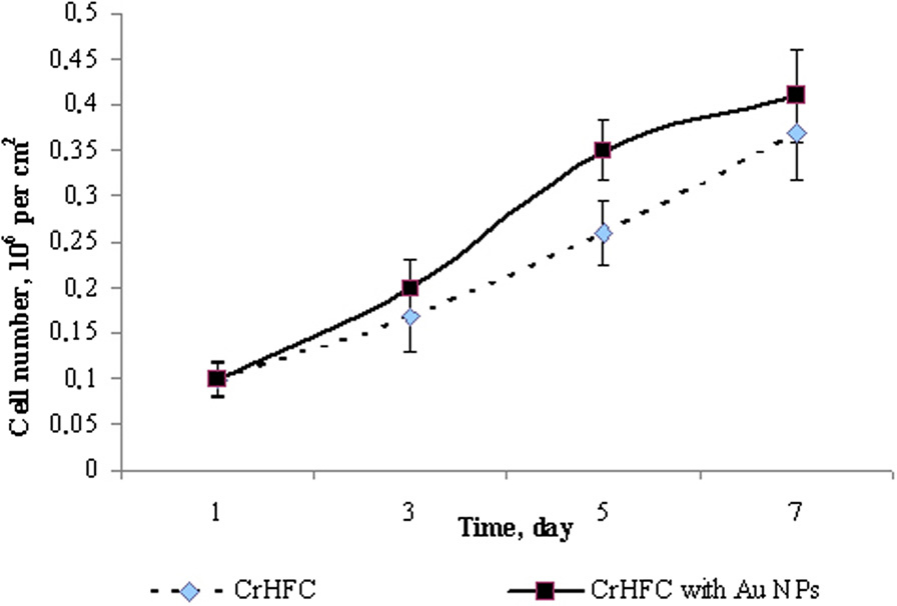
Proliferative activity of the CrHFC

Studying the growth dynamics, we establish that AuNPs in the investigated concentration render a stimulating effect on a proliferative ability of CrHFC cells (Fig. 1). This effect was manifested on day 3 of culturing and lasted until the end of the observation. The number of cells cultured with AuNPs increased by 1.35 times on day 5, but there was no statistical significance compared with the control group on day 7. At the end of culturing, 90 % confluence was reached in the control samples, and in those with an addition of 6 μg/ml AuNPs, the monolayer density was 95 %.

After the CrHFC application to the burns, the wound healing was monitored in all the groups within 21 days. In the animals with cell therapy, we marked a positive dynamics in burn wound regeneration. It should be noted that in the group with CrHFC therapy, burn healing occurred more slowly compared to the CrHFC with AuNP application. In both research groups, the burn wounds were clean, with no signs of inflammation in contrast to the control group, where the burns reduced due to the surface tension with purulent exudates in most of the cases. There were no wounds at the end of the experiment in the cases of the CrHFC application alone and with AuNPs, and on this place, a young skin appeared with quite complete epithelization.

The weight gain of the animals in the groups of therapy (CrHFC and CrHFC + AuNPs) has been noted to show a 1.2-fold significant increase in body weight relative to that in the control on day 21 (Table 2).

**Table 2:** Dynamics of body weight, g (*М* ± *m*, *n* = 5)

Group	Term, day			
	4	7	14	21
Control	186.7 ± 17.3	183.3 ± 15.5	206.7 ± 10.5	217.5 ± 20.5
CrHFC	173.3 ± 19.3	181.7 ± 20.5	199.3 ± 20.1	258.2 ± 24.8*
CrHFC + AuNPs	193.3 ± 22.6	200.1 ± 21.4	213.1 ± 20.3	260.2 ± 20.9*

*Statistical significance compared with the control group (*р* ≤ 0.05, *n* = 5)

The results of planimetric studies also showed that a decrease in the burn area was less with no complete wound healing in the control compared to the research groups during all observation. In the animals with cell therapy, the wound regeneration was stimulated, but the intensity of this process between the groups with CrHFC application alone and with AuNPs was different.

**Fig. 2:**
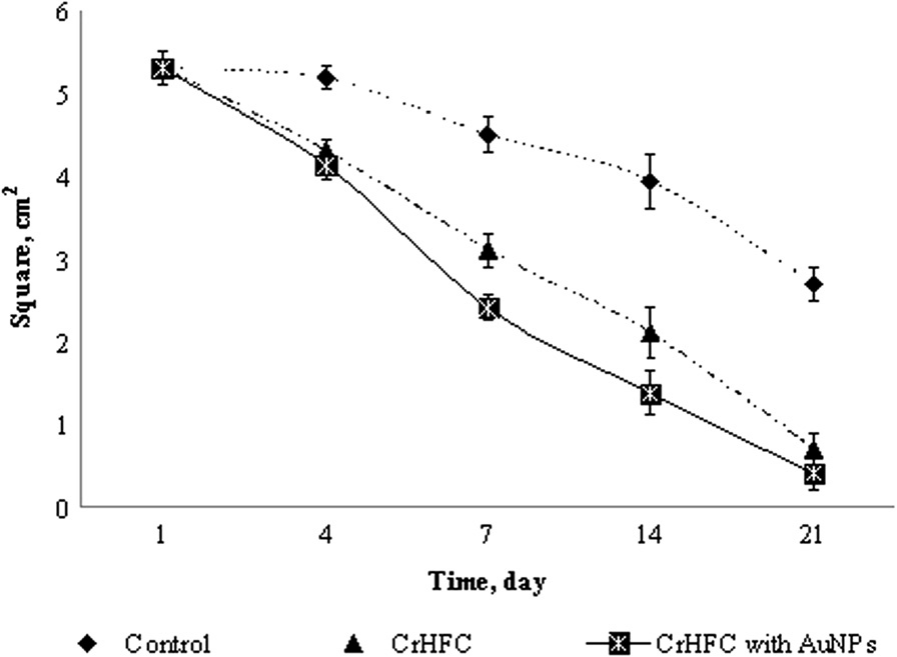
Dynamics of the wound area in rats after cell therapy

A decrease of the wound area was observed in the groups with CrHFC application alone and with AuNPs compared to those in the control on day 4 (Fig. 2). No significant difference was found between the research groups on this term.

The burn area decreased slightly in the animals of the control group on day 7. In groups with CrHFC application alone and with AuNPs, the area of the burn wound decreased by 1.5 and 1.9 times, respectively, compared to that of the control. The wounds in the animals from the research groups were clean; an eschar was not formed.

In the control group, the burn area contracted by 1.2 times on day 14 compared to the previous observation term. In the groups of the animals with CrHFC application alone and with AuNPs, the surface of the burn wound decreased by 1.9 and 2.9 times, respectively, compared to that of the control.

A positive tendency to reduce in the burn area was preserved in the control group on day 21. In the animals with cell therapy, the wounds were practically absent. A young skin with quite a complete epithelization was present at the burn place. It should be noted that differences between the research groups with cell therapy were significant on days 7 and 14, i.e., the wound healing process was faster in the case of CrHFC with AuNP application.

**Fig. 3:**
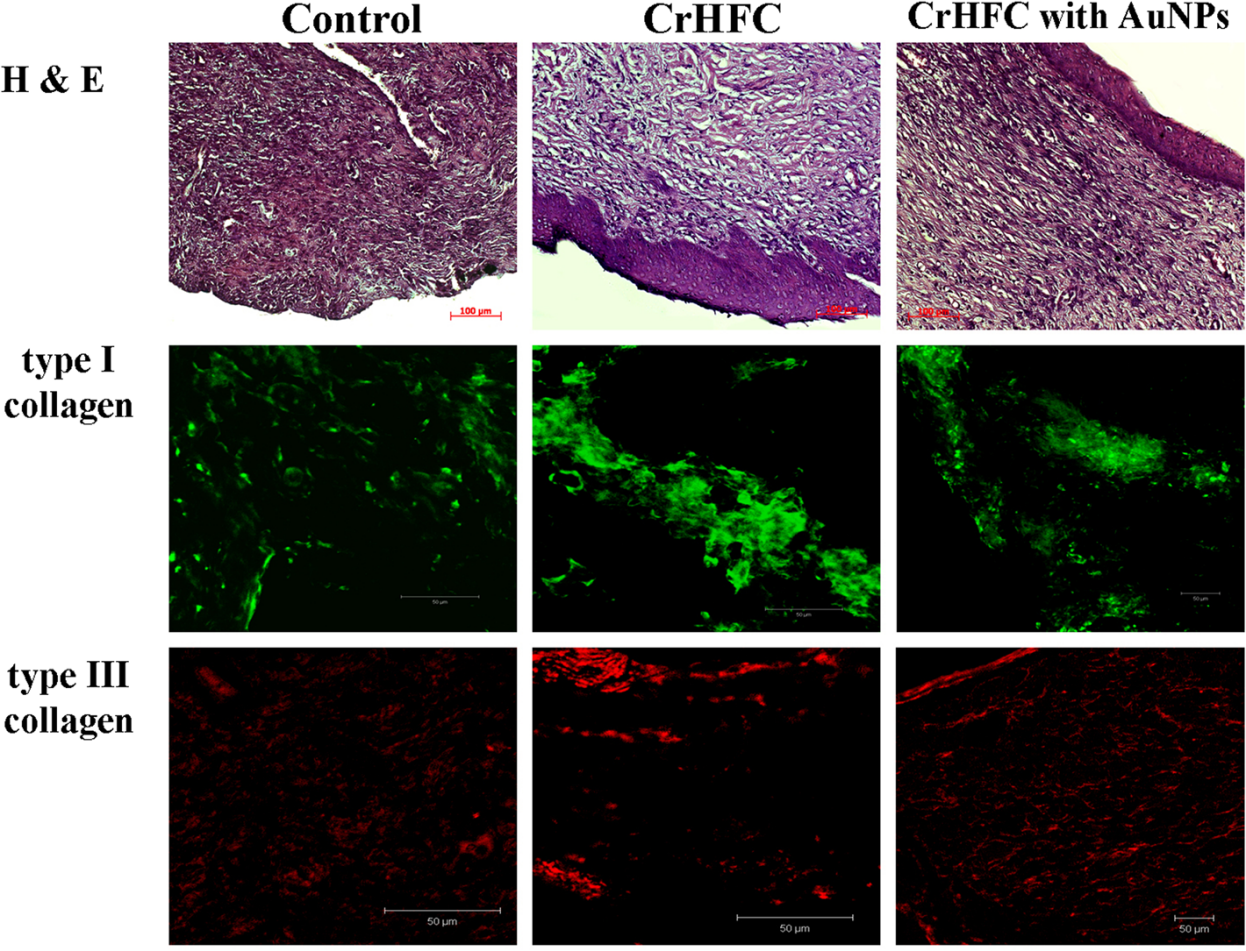
Burn wound of experimental groups on day 21

Microscopic examination of the skin specimens of control animals on day 21 of observation showed the signs of purulent burn wound (Fig. 3). There was an intense lymphoid infiltration in the central part of the wound restraining the formation of a granulation tissue. The epithelium regenerated from the edges with elimination in the center of the wounds. Collagen formation in deep derma layers along the wound edges occurred in the conditions of its infiltration by leukocytes. Bundles of collagen fibers had a random distribution and a low intensive fluorescence that covered the limited areas of the specimens stained with type I and III collagens (11.24 ± 2.91 % and 11.22 ± 1.84 %, respectively). The ratio of collagen type I/III was 1.0 that indicated an incomplete regenerative process.

A significant role in these changes possibly belongs to secondary deepening (secondary necrosis) that is a typical process for the burn wound. Thus, under the zone of primary necrosis, “a hidden injury” is forming immediately after exposure to damage factor where trophic changes lead to subsequent necrosis of tissues.

Histological examination of animal skin on day 21 after CrHFC application showed a reduction in the area and depth of the burns compared to the control (Fig. 3). Formed granulation tissue was moderately infiltrated by lymphocytes. There was determined a young connective tissue on the border with the area of wound defect that had an unordered collagen and elastic fibers among which quite a big number of fibroblast-like cells was located. A laminated epithelium was growing from the edges of the wound and began to differentiate into layers. The epithelium exfoliated and eliminated only in separate minor areas in the center of the wounds. Immune fluorescence of type I collagen was intense and covered 52.06 ± 11.12 % of the specimens. The areas of type III collagen positive fluorescence occupied 23.63 ± 8.61 % and were intense along the edges of the specimens. The ratio of collagen type I/III was 2.2. In general, this research group showed a more active course of regenerative processes than the control group.

Microscopic study of wounds on day 21 after CrHFC with AuNP application showed a complete epithelization of burns. Lymphoid infiltration was not detected. Wound defect was completely replaced by young connective tissue in which the collagen and elastic fibers were oriented orderly with a significant number of fibroblast-like cells between them. It should be noted that the newly formed connective tissue was penetrated by microvessels which is very important for wound healing due to the activation of metabolic processes. Over the area of the wound defect, a newly formed epithelial layer was slightly thinner than in the area surrounding the wound. This could be due to the stretching of the epithelium as the newly formed connective tissue grew up under it. Immune fluorescence of type I and III collagens was clear, regular, evenly distributed, and covered 30.36 ± 8.2 % and 13.91 ± 5.2 % of the area, respectively. The ratio of collagen type I/III was 2.18. These results showed an active course of regenerative processes as well as in the group with CrHFC application.

## Discussion

Rapid healing of dermatological wounds is of vital importance in preventing infection and reducing post-treatment side effects. Topical applications of antioxidant agents on cutaneous wounds have attracted much attention. AuNPs were shown to have antioxidative effects and could be helpful in wound healing. Leu et al. [14] reported that topical AuNP application with epigallocatechin gallate and α-lipoic acid significantly accelerated mouse cutaneous wound healing through anti-inflammatory and antioxidation effects.

The other authors also reported of the therapeutic effects of AuNPs coated on a hydrocolloid membrane (HCM) due to the stimulation of antioxidant effects as well as the synergistic regulation of angiogenesis and connective tissue formation [15]. They showed a significant decrease in matrix metalloproteinase-1 expression and transforming growth factor concentration in the phytochemically stabilized AuNP-HCM-treated group on day 5. Wound tissue applied with the phytochemically stabilized AuNP-HCM showed an enhancement of vascular endothelial growth factor, angiopoietin 1, and angiopoietin 2 expression. Furthermore, the activity of superoxide dismutases and collagen expression was significantly increased in the skin tissue of the phytochemically stabilized AuNP-HCM-treated group.

Wound healing is a complex process that involves a plethora of cells, proteins, enzymes, cytokines, hormones, and ions. One of the central components of extracellular matrix products during a wound healing is collagens. However, the production of collagen can be a double-edged sword. On the one hand, it is necessary for wound healing; On the other hand, excess deposition of collagen can result in scarring [16]. Many clinical problems are associated with an excessive scar formation, for example, keloids and hypertrophic scars in the skin, tendon adhesions, transmission blockage following nerve injury, scleroderma, Crohn’s disease, esophageal and urethral structures, capsules around breast implants, liver cirrhosis, atherosclerosis, and fibrotic non-union joint [17]. Therefore, the appropriate expression of collagen is required for an ideal wound healing. Our findings indicate that CrHFC with AuNPs accelerate wound healing by increasing cell proliferation and subsequent regulation of collagen synthesis/degradation as well as alteration of type I and III collagen composition in the injury site.

The study of the impact of CrHFC application alone and with AuNPs on the burn surface showed that both investigated cell preparations stimulated the healing of burn wounds. The results of clinical and planimetric examinations of burn wounds were in conformity to histological findings. The use of CrHFC cells alone had a less pronounced effect than CrHFC with AuNPs, that was reflected in slower recovery of burns and mild lymphocyte infiltration of granulation tissue, while in the case of CrHFC application with AuNPs an almost complete epithelization of the wound was observed. The results are probably related to the unique structure and antimicrobial properties of AuNPs. Immunofluorescent analysis indicated that the use of CrHFC with AuNPs accelerated synthetic processes in the skin and contributed to the restoration of type I collagen content on day 21 after application.

## Conclusions

This study demonstrated that CrHFC with gold nanoparticles contributed to the healing of the burn and promoted the activation of regenerative processes in the damaged tissues that manifested in increasing the burn contraction rate and restoration of the skin histological structure and type I and III collagen content on post-treatment day 21. Although only one type of NPs has been used, our results suggest that this novel methodology has major implications in the future of nano- and biotechnologies strategies in the treatment of wounds.
